# The genome sequence of the pond mud snail, *Omphiscola glabra *(O.F.Müller, 1774)

**DOI:** 10.12688/wellcomeopenres.24266.1

**Published:** 2025-05-20

**Authors:** Katarzyna Ruta, Helen R Taylor, Wellcome Sanger Institute Tree of Life Management, Samples and Laboratory team, Wellcome Sanger Institute Scientific Operations: Sequencing Operations, Wellcome Sanger Institute Tree of Life Core Informatics team, Tree of Life Core Informatics collective, Darwin Tree of Life Consortium

**Affiliations:** 1 Royal Zoological Society of Scotland, Edinburgh, Scotland, UK

**Keywords:** Omphiscola glabra, pond mud snail, genome sequence, chromosomal, Mollusca

## Abstract

We present a genome assembly from a specimen of *Omphiscola glabra* (pond mud snail; Mollusca; Gastropoda; Lymnaeidae). The genome sequence has a total length of 744.78 megabases. Most of the assembly (99.95%) is scaffolded into 18 chromosomal pseudomolecules. The mitochondrial genome has also been assembled, with a length of 13.78 kilobases. Gene annotation of this assembly on Ensembl identified 18,317 protein-coding genes.

## Species taxonomy

Eukaryota; Opisthokonta; Metazoa; Eumetazoa; Bilateria; Protostomia; Spiralia; Lophotrochozoa; Mollusca; Gastropoda; Heterobranchia; Euthyneura; Panpulmonata; Hygrophila; Lymnaeoidea; Lymnaeidae; *Omphiscola*; *Omphiscola glabra* (O.F.Müller, 1774) (NCBI:txid161078)

## Background

**Figure 1.**
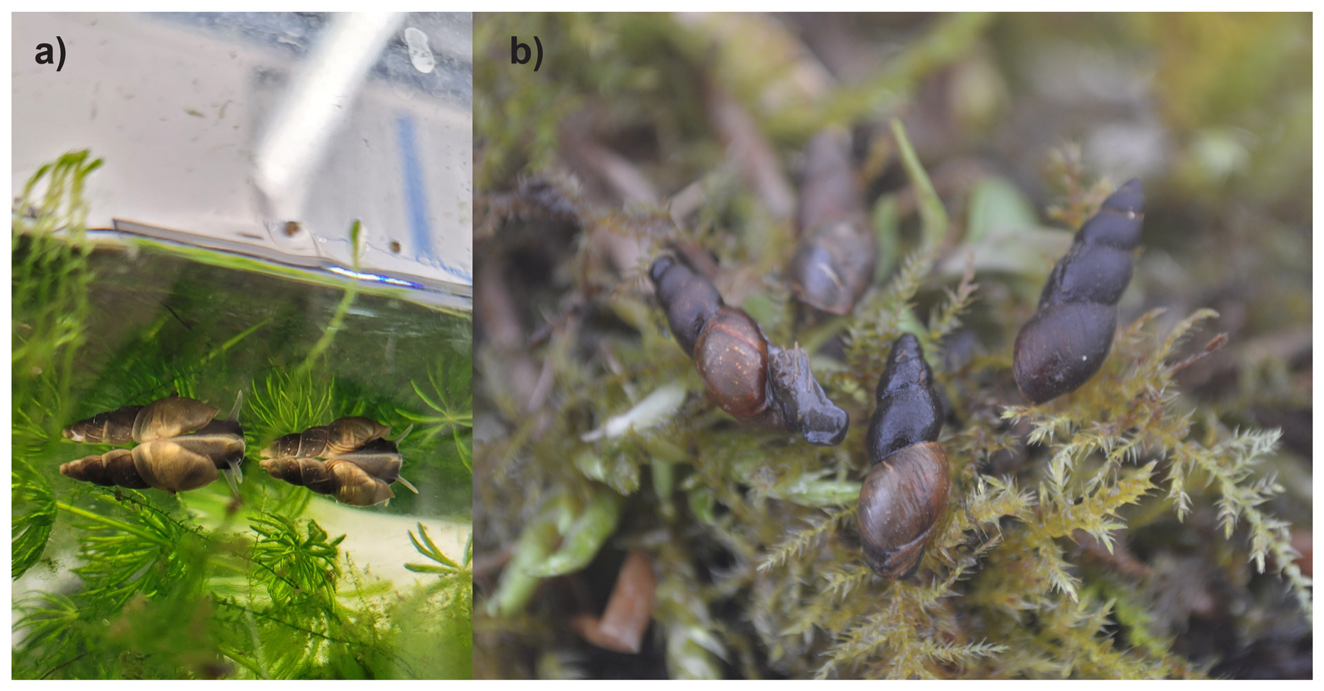
Photographs of *Omphiscola glabra* (not the specimen used for genome sequencing). **a**) adult individuals in the conservation breeding facility at RZSS’ Highland Wildlife Park site. **B**) adult individuals found during a survey of one of the reintroduction release sites for the species in Red Moss of Balerno near Edinburgh.

The pond mud snail, or slender pond snail, ( *Omphiscola glabra*) is a small species of freshwater mollusc ( Figure 1). Once widespread across the acidic lowlands of the UK, the species has undergone a significant decline since 1960 ( Kerney, 1999) and is considered nationally scarce ( Seddon *et al.*, 2014). In Scotland it is currently restricted to a handful of sites (seven in total according to the most recent surveys ( Buglife, 2019). The main reasons for the pond mud snail's decline include habitat loss and degradation, farmland drainage, and pollution ( Buglife, 2019; Dreyfuss *et al.*, 2018; Macadam & Baker, 2005) as well as enlargement of temporary ponds into permanent ones ( Rowson *et al.*, 2021). In addition, these snails are not able to move between habitats easily and there is a general lack of knowledge and awareness of the species. The pond mud snail is a UK Biodiversity Action Plan species, and is listed on the Scottish Biodiversity List. On a global scale, the species is found in several countries in Western Europe and Scandinavia and is categorised as Near Threatened on the IUCN Red List of Threatened Species ( Buglife, 2024; Prié *et al.*, 2011).

Intensive conservation efforts for pond mud snails in Scotland have been ongoing since 2017. From 2017 to 2019, the Buglife-led Marvellous Mud Snails project worked to create a healthier and more resilient pond mud snail population through public engagement, habitat creation and *ex-situ* conservation breeding ( Buglife, 2024). Through partnership with Royal Zoological Society of Scotland (RZSS) a conservation breeding for release population was established at RZSS Edinburgh Zoo using founders from a rediscovered population in Bavelaw Marsh, Edinburgh. Successful breeding has been achieved by RZSS every year since 2017. In 2018, 80 pond mud snails bred at Edinburgh Zoo were released across two small ponds at the Red Moss of Balerno reserve site (close to the Bavelaw Marsh source site) by Buglife and RZSS. Post-release monitoring did not detect the snails at either release site for several years, until April 2023 when the species was recorded at one of the release sites, where it has been found regularly ever since. Since 2024, the RZSS breeding programme has been relocated to the organisation’s Highland Wildlife Park site, where successful breeding continues. Further reintroduction releases to sites in the vicinity of the Bavelaw Marsh source population are being planned for 2025.

To date, there has been very little population genetic work conducted on pond mud snails. The one study we are aware of used allozymes to investigate the genetic diversity of a population of this species in central France ( Hurtrez-Boussès *et al.*, 2005). A genetic study on population structure and differentiation between the seven extant pond mud snail populations in Scotland would be extremely useful for translocation planning, allowing assessment of the risks and benefits of translocating individuals between sites and of mixing individuals from different Scottish populations in the conservation breeding programme. The genome sequence presented here is a useful step towards conducting such analyses and improving conservation planning for pond mud snails in the UK and beyond. Here we present a chromosomal-level genome sequence for *Omphiscola glabra*, based on a specimen from Edinburgh Zoo, Scotland, United Kingdom.

## Genome sequence report

### Sequencing data

The genome of a specimen of *Omphiscola glabra* ( Figure 1) was sequenced using Pacific Biosciences single-molecule HiFi long reads, generating 42.82 Gb from 4.56 million reads, which were used to assemble the genome. GenomeScope analysis estimated the haploid genome size at 684.75 Mb, with a heterozygosity of 0.45% and repeat content of 22.38%. These estimates guided expectations for the assembly. Based on the estimated genome size, the sequencing data provided approximately 57 coverage. Hi-C sequencing produced 223.91 Gb from 1,482.83 million reads, and was used to scaffold the assembly. RNA sequencing data were also generated and are available in public sequence repositories. Table 1 summarises the specimen and sequencing details.

**Table 1.** Specimen and sequencing data for *Omphiscola glabra*.

Project information			
**Study title**	Omphiscola glabra (pond mud snail)		
**Umbrella BioProject**	PRJEB73399		
**Species**	*Omphiscola glabra*		
**BioSpecimen**	SAMEA9654424		
**NCBI taxonomy ID**	161078		
Specimen information			
**Technology**	**ToLID**	**BioSample accession**	**Organism part**
**PacBio long read sequencing**	xgOmpGlab2	SAMEA9654437	whole organism
**Hi-C sequencing**	xgOmpGlab5	SAMEA9654440	whole organism
**RNA sequencing**	xgOmpGlab6	SAMEA9654441	whole organism
Sequencing information			
**Platform**	**Run accession**	**Read count**	**Base count (Gb)**
**Hi-C Illumina NovaSeq 6000**	ERR12723465	7.57e+08	114.37
**Hi-C Illumina NovaSeq X**	ERR12743801	7.25e+08	109.53
**PacBio Revio**	ERR12721052	4.56e+06	42.82
**RNA Illumina NovaSeq 6000**	ERR12723466	6.36e+07	9.61

### Assembly statistics

The primary haplotype was assembled, and contigs corresponding to an alternate haplotype were also deposited in INSDC databases. The assembly was improved by manual curation, which corrected 17 misjoins or missing joins. These interventions decreased the scaffold count by 9.76%. The final assembly has a total length of 744.78 Mb in 36 scaffolds, with 289 gaps, and a scaffold N50 of 41.46 Mb ( Table 2).

**Table 2.** Genome assembly data for *Omphiscola glabra*.

Genome assembly		
Assembly name	xgOmpGlab2.1	
Assembly accession	GCA_964017045.1	
*Alternate haplotype accession*	*GCA_964017015.1*	
Assembly level for primary assembly	chromosome	
Span (Mb)	744.78	
Number of contigs	325	
Number of scaffolds	36	
Longest scaffold (Mb)	86.24	
Assembly metric	Measure	*Benchmark*
Contig N50 length	3.67 Mb	*≥ 1 Mb*
Scaffold N50 length	41.46 Mb	*= chromosome N50*
Consensus quality (QV)	Primary: 60.1; alternate: 52.8; combined: 54.8	*≥ 40*
*k*-mer completeness	Primary: 97.61%; alternate: 40.05%; combined: 98.93%	*≥ 95%*
BUSCO *	C:93.2%[S:92.5%,D:0.7%], F:1.9%,M:4.8%,n:5,295	*S > 90%; D < 5%*
Percentage of assembly mapped to chromosomes	99.95%	*≥ 90%*
Organelles	Mitochondrial genome: 13.78 kb	*complete single alleles*

* BUSCO scores based on the mollusca_odb10 BUSCO set using version 5.5.0. C = complete [S = single copy, D = duplicated], F = fragmented, M = missing, n = number of orthologues in comparison.

**Figure 2.**
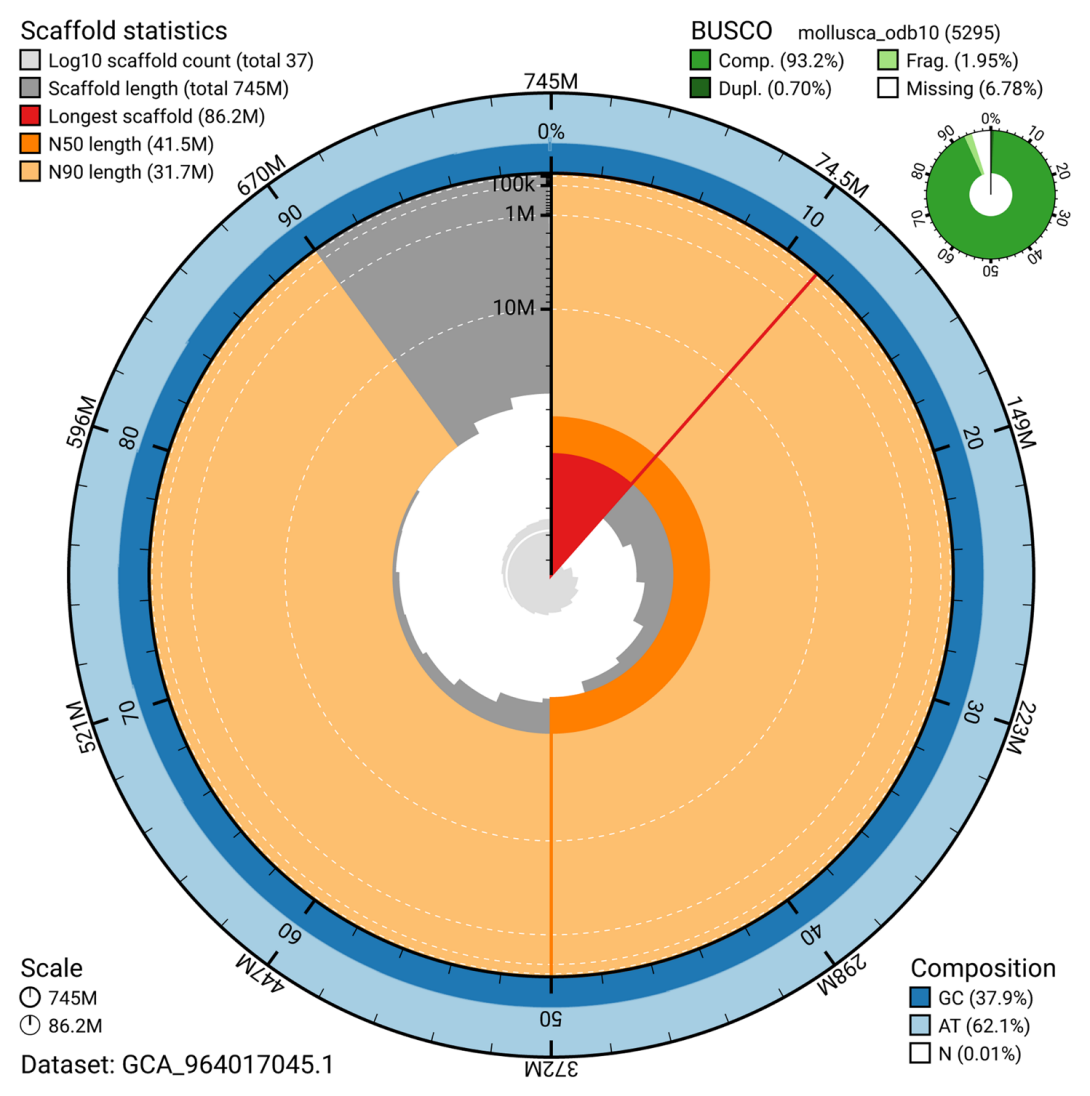
Genome assembly of *Omphiscola glabra*, xgOmpGlab2.1: metrics. The BlobToolKit snail plot provides an overview of assembly metrics and BUSCO gene completeness. The circumference represents the length of the whole genome sequence, and the main plot is divided into 1,000 bins around the circumference. The outermost blue tracks display the distribution of GC, AT, and N percentages across the bins. Scaffolds are arranged clockwise from longest to shortest and are depicted in dark grey. The longest scaffold is indicated by the red arc, and the deeper orange and pale orange arcs represent the N50 and N90 lengths. A light grey spiral at the centre shows the cumulative scaffold count on a logarithmic scale. A summary of complete, fragmented, duplicated, and missing BUSCO genes in the set is presented at the top right. An interactive version of this figure is available at https://blobtoolkit.genomehubs.org/view/GCA_964017045.1/dataset/GCA_964017045.1/snail.

**Figure 3.**
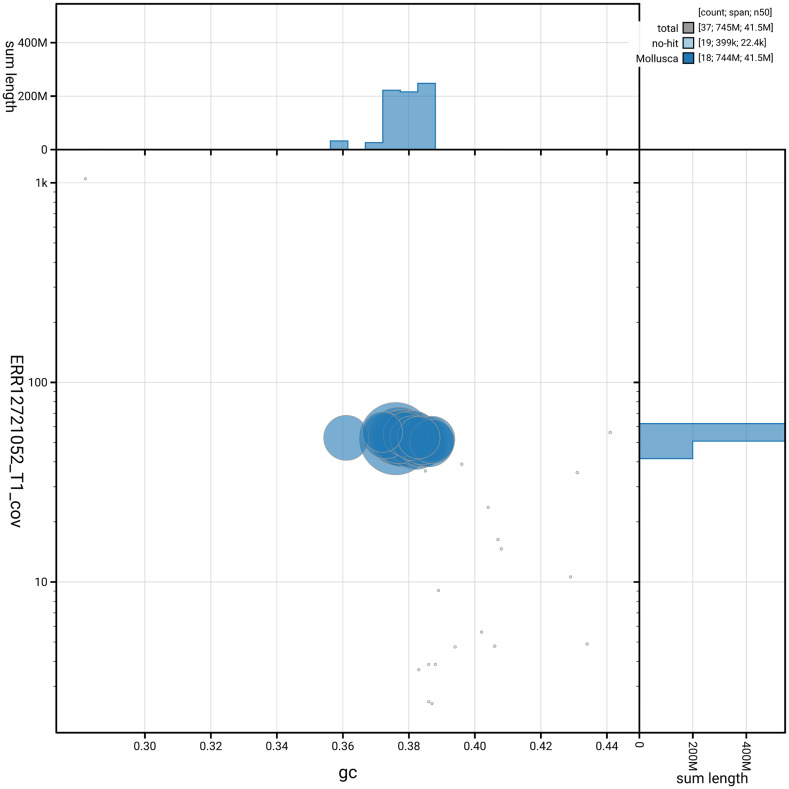
Genome assembly of *Omphiscola glabra*, xgOmpGlab2.1: BlobToolKit GC-coverage plot. Blob plot showing sequence coverage (vertical axis) and GC content (horizontal axis). The circles represent scaffolds, with the size proportional to scaffold length and the colour representing phylum membership. The histograms along the axes display the total length of sequences distributed across different levels of coverage and GC content. An interactive version of this figure is available at https://blobtoolkit.genomehubs.org/view/GCA_964017045.1/dataset/GCA_964017045.1/blob.

**Figure 4.**
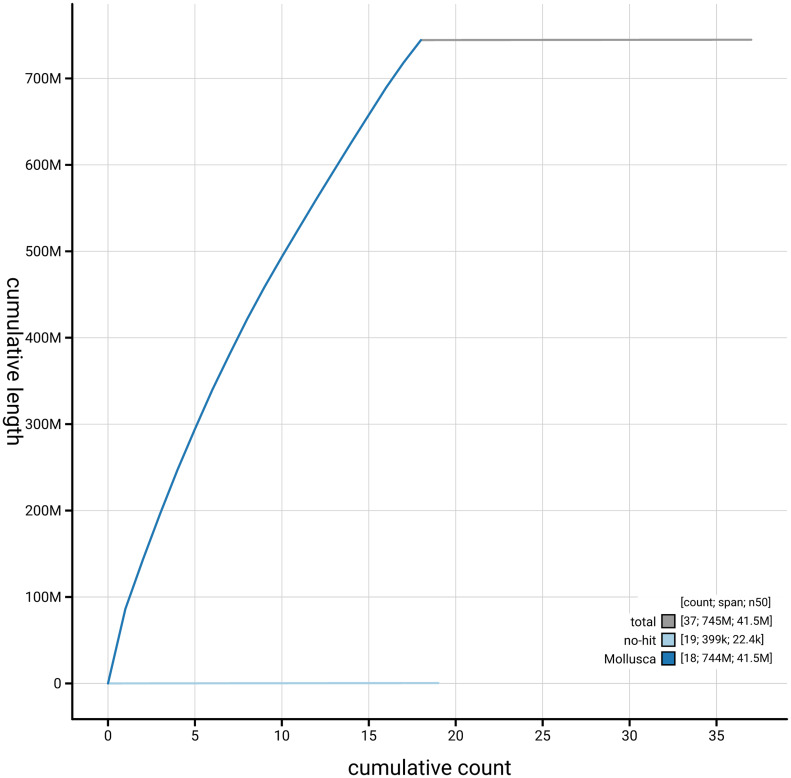
Genome assembly of *Omphiscola glabra,* xgOmpGlab2.1: BlobToolKit cumulative sequence plot. The grey line shows cumulative length for all scaffolds. Coloured lines show cumulative lengths of scaffolds assigned to each phylum using the buscogenes taxrule. An interactive version of this figure is available at https://blobtoolkit.genomehubs.org/view/GCA_964017045.1/dataset/GCA_964017045.1/cumulative.

The snail plot in Figure 2 provides a summary of the assembly statistics, indicating the distribution of scaffold lengths and other assembly metrics. Figure 3 shows the distribution of scaffolds by GC proportion and coverage. Figure 4 presents a cumulative assembly plot, with separate curves representing different scaffold subsets assigned to various phyla, illustrating the completeness of the assembly.

**Figure 5.**
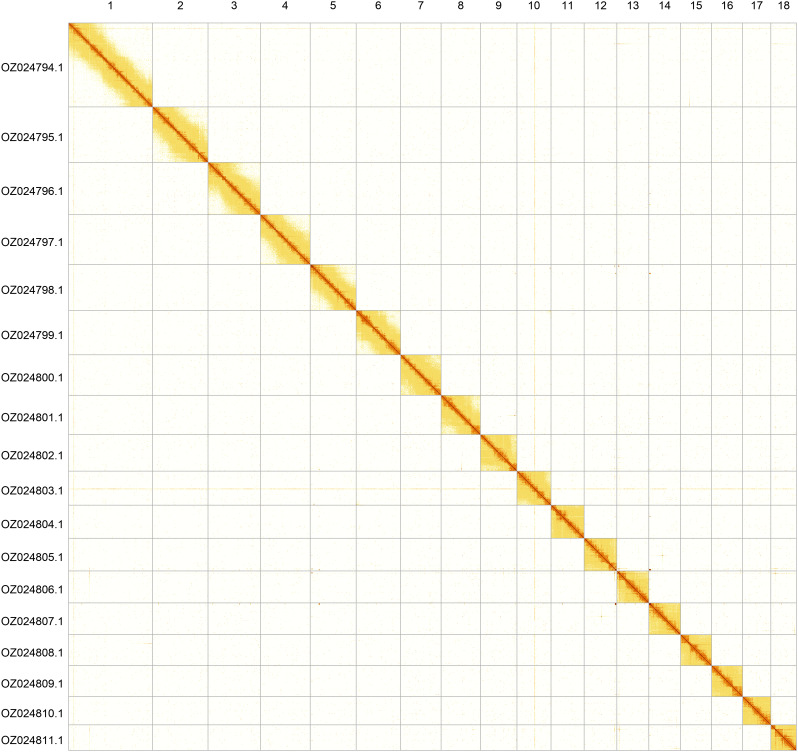
Genome assembly of *Omphiscola glabra*. Hi-C contact map of the xgOmpGlab2.1 assembly, generated using PretextSnapshot. Chromosomes are shown in order of size and labelled with chromosome numbers (top) and chromosome accession numbers (left).

Most of the assembly sequence (99.95%) was assigned to 18 chromosomal-level scaffolds. These chromosome-level scaffolds, confirmed by Hi-C data, are named according to size ( Figure 5; Table 3).

**Table 3.** Chromosomal pseudomolecules in the genome assembly of *Omphiscola glabra*, xgOmpGlab2.

INSDC accession	Name	Length (Mb)	GC%
OZ024794.1	1	86.24	37.5
OZ024795.1	2	56.58	37.5
OZ024796.1	3	53.61	37.5
OZ024797.1	4	50.82	38
OZ024798.1	5	47.08	38
OZ024799.1	6	45.37	37.5
OZ024800.1	7	41.46	38.5
OZ024801.1	8	40.25	38.5
OZ024802.1	9	37.24	38.5
OZ024803.1	10	34.91	38.5
OZ024804.1	11	33.83	37.5
OZ024805.1	12	33.53	38.5
OZ024806.1	13	32.65	38
OZ024807.1	14	32.5	36
OZ024808.1	15	31.76	38
OZ024809.1	16	31.69	38.5
OZ024810.1	17	28.79	38.5
OZ024811.1	18	26.09	37
OZ024812.1	MT	0.01	28

The mitochondrial genome was also assembled. This sequence is included as a contig in the multifasta file of the genome submission and as a standalone record.

### Assembly quality metrics

The estimated Quality Value (QV) and *k*-mer completeness metrics, along with BUSCO completeness scores, were calculated for each haplotype and the combined assembly. The QV reflects the base-level accuracy of the assembly, while *k*-mer completeness indicates the proportion of expected *k*-mers identified in the assembly. BUSCO scores provide a measure of completeness based on benchmarking universal single-copy orthologues.

The combined primary and alternate assemblies achieve an estimated QV of 54.8. The *k*-mer recovery for the primary haplotype is 97.61%, and for the alternate haplotype 40.05%; the combined primary and alternate assemblies have a *k*-mer recovery of 98.93%. BUSCO v.5.5.0 analysis using the mollusca_odb10 reference set ( *n* = 5,295) identified 93.2% of the expected gene set (single = 92.5%, duplicated = 0.7%).

Table 2 provides assembly metric benchmarks adapted from Rhie *et al.* (2021) and the Earth BioGenome Project Report on Assembly Standards September 2024. The assembly achieves the EBP reference standard of **6.C.Q60**.

## Genome annotation report

The *Omphiscola glabra* genome assembly (GCA_964017045.1) was annotated externally by Ensembl at the European Bioinformatics Institute (EBI). This annotation includes 31,314 transcribed mRNAs from 18,317 protein-coding and 4,006 non-coding genes. The average transcript length is 14,433.37 bp. There are 1.40 coding transcripts per gene and 8.37 exons per transcript. For further information about the annotation, please refer to https://beta.ensembl.org/species/bdcfdb5c-5307-4daa-b22b-f51d688ae687.

## Methods

### Sample acquisition and DNA barcoding

Specimens of *Omphiscola glabra* were collected from Edinburgh Zoo, Scotland, United Kingdom (latitude 55.9424, longitude –3.2686) on 2021-06-10. The specimen used for genome sequencing was specimen ID SAN0001372 (ToLID xgOmpGlab2), a second specimen was used for Hi-C sequencing (specimen ID SAN0001375, ToLID xgOmpGlab5), and a third was used for RNA sequencing (specimen ID SAN0001376, ToLID xgOmpGlab6). All specimens were collected and identified by Helen Taylor (RZSS) and placed in a liquid nitrogen dry shipper immediately after death.

Metadata collection for samples adhered to the Darwin Tree of Life project standards described by Lawniczak *et al.* (2022).

### Nucleic acid extraction

The workflow for high molecular weight (HMW) DNA extraction at the Wellcome Sanger Institute (WSI) Tree of Life Core Laboratory includes a sequence of procedures: sample preparation and homogenisation, DNA extraction, fragmentation and purification. Detailed protocols are available on protocols.io ( Denton *et al.*, 2023). The xgOmpGlab2 sample was prepared for DNA extraction by weighing and dissecting it on dry ice ( Jay *et al.*, 2023). Tissue from the whole organism was cryogenically disrupted using the Covaris cryoPREP ^®^ Automated Dry Pulverizer ( Narváez-Gómez *et al.*, 2023). HMW DNA was extracted using the Automated MagAttract v2 protocol ( Oatley *et al.*, 2023a). DNA was sheared into an average fragment size of 12–20 kb in a Megaruptor 3 system ( Bates *et al.*, 2023). Sheared DNA was purified by solid-phase reversible immobilisation, using AMPure PB beads to eliminate shorter fragments and concentrate the DNA ( Oatley *et al.*, 2023b). The concentration of the sheared and purified DNA was assessed using a Nanodrop spectrophotometer and Qubit Fluorometer using the Qubit dsDNA High Sensitivity Assay kit. Fragment size distribution was evaluated by running the sample on the FemtoPulse system.

RNA was extracted from whole organism tissue of xgOmpGlab6 in the Tree of Life Laboratory at the WSI using the RNA Extraction: Automated MagMax™ *mir*Vana protocol ( do Amaral *et al.*, 2023). The RNA concentration was assessed using a Nanodrop spectrophotometer and a Qubit Fluorometer using the Qubit RNA Broad-Range Assay kit. Analysis of the integrity of the RNA was done using the Agilent RNA 6000 Pico Kit and Eukaryotic Total RNA assay.

### Hi-C sample preparation and crosslinking

Hi-C data were generated from the whole organism of the xgOmpGlab5 sample using the Arima-HiC v2 kit (Arima Genomics) with 20–50 mg of frozen tissue (stored at –80 °C). As per manufacturer’s instructions, tissue was fixed, and the DNA crosslinked using a TC buffer with 22% formaldehyde concentration, and a final formaldehyde concentration of 2%. The tissue was then homogenised using the Diagnocine Power Masher-II. The crosslinked DNA was digested using a restriction enzyme master mix, then biotinylated and ligated. A clean up was performed with SPRIselect beads prior to library preparation. DNA concentration was quantified using the Qubit Fluorometer v4.0 (Thermo Fisher Scientific) and Qubit HS Assay Kit, and sample biotinylation percentage was estimated using the Arima-HiC v2 QC beads.

### Library preparation and sequencing

Library preparation and sequencing were performed at the WSI Scientific Operations core.

***PacBio HiFi***

At a minimum, samples were required to have an average fragment size exceeding 8 kb and a total mass over 400 ng to proceed to the low-input SMRTbell Prep Kit 3.0 protocol (Pacific Biosciences), depending on genome size and sequencing depth required. Libraries were prepared using the SMRTbell Prep Kit 3.0 as per the manufacturer's instructions. The kit includes the reagents required for end repair/A-tailing, adapter ligation, post-ligation SMRTbell bead cleanup, and nuclease treatment. Size-selection and clean-up were carried out using diluted AMPure PB beads (Pacific Biosciences). DNA concentration was quantified using the Qubit Fluorometer v4.0 (ThermoFisher Scientific) with Qubit 1X dsDNA HS assay kit and the final library fragment size analysis was carried out using the Agilent Femto Pulse Automated Pulsed Field CE Instrument (Agilent Technologies) and the gDNA 55kb BAC analysis kit.

Samples were sequenced on a Revio instrument (Pacific Biosciences, California, USA). Prepared libraries were normalised to 2 nM, and 15 μL was used for making complexes. Primers were annealed and polymerases were bound to create circularised complexes according to manufacturer’s instructions. The complexes were purified with the 1.2X clean up with SMRTbell beads. The purified complexes were then diluted to the Revio loading concentration (in the range 200–300 pM), and spiked with a Revio sequencing internal control. Samples were sequenced on Revio 25M SMRT cells (Pacific Biosciences, California, USA). The SMRT link software, a PacBio web-based end-to-end workflow manager, was used to set-up and monitor the run, as well as perform primary and secondary analysis of the data upon completion.

***Hi-C***

For Hi-C library preparation, the biotinylated DNA constructs were fragmented using a Covaris E220 sonicator and size-selected to 400–600 bp using SPRISelect beads. DNA was then enriched using Arima-HiC v2 Enrichment beads. The NEBNext Ultra II DNA Library Prep Kit (New England Biolabs) was used for end repair, A-tailing, and adapter ligation, following a modified protocol in which library preparation is carried out while the DNA remains bound to the enrichment beads. PCR amplification was performed using KAPA HiFi HotStart mix and custom dual-indexed adapters (Integrated DNA Technologies) in a 96-well plate format. Depending on sample concentration and biotinylation percentage determined at the crosslinking stage, samples were amplified for 10–16 PCR cycles. Post-PCR clean-up was carried out using SPRISelect beads. The libraries were quantified using the Accuclear Ultra High Sensitivity dsDNA Standards Assay kit (Biotium) and normalised to 10 ng/μL before sequencing. Hi-C sequencing was performed on the Illumina NovaSeq X instrument using 150 bp paired-end reads.

***RNA***

Libraries were prepared using the NEBNext ^®^ Ultra™ II Directional RNA Library Prep Kit for Illumina (New England Biolabs), following the manufacturer’s instructions. Poly(A) mRNA in the total RNA solution was isolated using oligo(dT) beads, converted to cDNA, and uniquely indexed; 14 PCR cycles were performed. Libraries were size-selected to produce fragments between 100–300 bp. Libraries were quantified, normalised, pooled to a final concentration of 2.8 nM, and diluted to 150 pM for loading. Sequencing was carried out on the Illumina NovaSeq 6000 instrument, generating 150-bp paired-end reads.

### Genome assembly, curation and evaluation

***Assembly***

Prior to assembly of the PacBio HiFi reads, a database of *k*-mer counts ( *k* = 31) was generated from the filtered reads using FastK. GenomeScope2 ( Ranallo-Benavidez *et al.*, 2020) was used to analyse the *k*-mer frequency distributions, providing estimates of genome size, heterozygosity, and repeat content.

The HiFi reads were first assembled using Hifiasm ( Cheng *et al.*, 2021) with the --primary option. Haplotypic duplications were identified and removed using purge_dups ( Guan *et al.*, 2020). The Hi-C reads ( Rao *et al.*, 2014) were mapped to the primary contigs using bwa-mem2 ( Vasimuddin *et al.*, 2019), and the contigs were scaffolded using YaHS ( Zhou *et al.*, 2023) using the --break option for handling potential misassemblies. The scaffolded assemblies were evaluated using Gfastats ( Formenti *et al.*, 2022), BUSCO ( Manni *et al.*, 2021) and MERQURY.FK ( Rhie *et al.*, 2020).

The mitochondrial genome was assembled using MitoHiFi ( Uliano-Silva *et al.*, 2023), which runs MitoFinder ( Allio *et al.*, 2020) and uses these annotations to select the final mitochondrial contig and to ensure the general quality of the sequence.

***Assembly curation***

The assembly was decontaminated using the Assembly Screen for Cobionts and Contaminants (ASCC) pipeline. Flat files and maps used in curation were generated via the TreeVal pipeline ( Pointon *et al.*, 2023). Manual curation was conducted primarily in PretextView ( Harry, 2022) and HiGlass ( Kerpedjiev *et al.*, 2018), with additional insights provided by JBrowse2 ( Diesh *et al.*, 2023). Scaffolds were visually inspected and corrected as described by Howe *et al.* (2021). Any identified contamination, missed joins, and mis-joins were amended, and duplicate sequences were tagged and removed. The curation process is documented at https://gitlab.com/wtsi-grit/rapid-curation. PretextSnapshot was used to generate a Hi-C contact map of the final assembly.

***Assembly quality assessment***

The Merqury.FK tool ( Rhie *et al.*, 2020), run in a Singularity container ( Kurtzer *et al.*, 2017), was used to evaluate *k*-mer completeness and assembly quality for the primary and alternate haplotypes using the *k*-mer databases ( *k* = 31) computed prior to genome assembly. The analysis outputs included assembly QV scores and completeness statistics.

The genome was analysed in the blobtoolkit pipeline, a Nextflow ( Di Tommaso *et al.*, 2017) port of the previous Snakemake Blobtoolkit pipeline ( Challis *et al.*, 2020). It aligns the PacBio reads in SAMtools ( Danecek *et al.*, 2021) and minimap2 ( Li, 2018) and generates coverage tracks for regions of fixed size. In parallel, it queries the GoaT database ( Challis *et al.*, 2023) to identify all matching BUSCO lineages to run BUSCO ( Manni *et al.*, 2021). For the three domain-level BUSCO lineages, the pipeline aligns the BUSCO genes to the UniProt Reference Proteomes database ( Bateman *et al.*, 2023) with DIAMOND blastp ( Buchfink *et al.*, 2021). The genome is also divided into chunks according to the density of the BUSCO genes from the closest taxonomic lineage, and each chunk is aligned to the UniProt Reference Proteomes database using DIAMOND blastx. Genome sequences without a hit are chunked using seqtk and aligned to the NT database with blastn ( Altschul *et al.*, 1990). The blobtools suite combines all these outputs into a blobdir for visualisation.

The blobtoolkit pipeline was developed using nf-core tooling ( Ewels *et al.*, 2020) and MultiQC ( Ewels *et al.*, 2016), relying on the Conda package manager, the Bioconda initiative ( Grüning *et al.*, 2018), the Biocontainers infrastructure ( da Veiga Leprevost *et al.*, 2017), as well as the Docker ( Merkel, 2014) and Singularity ( Kurtzer *et al.*, 2017) containerisation solutions.

Table 4 contains a list of relevant software tool versions and sources.

**Table 4.** Software tools: versions and sources.

Software tool	Version	Source
BLAST	2.14.0	ftp://ftp.ncbi.nlm.nih.gov/blast/executables/blast+/
BlobToolKit	4.3.9	https://github.com/blobtoolkit/blobtoolkit
BUSCO	5.5.0	https://gitlab.com/ezlab/busco
bwa-mem2	2.2.1	https://github.com/bwa-mem2/bwa-mem2
DIAMOND	2.1.8	https://github.com/bbuchfink/diamond
fasta_windows	0.2.4	https://github.com/tolkit/fasta_windows
FastK	666652151335353eef2fcd58880bcef5bc2928e1	https://github.com/thegenemyers/FASTK
GenomeScope2.0	2.0.1	https://github.com/tbenavi1/genomescope2.0
Gfastats	1.3.6	https://github.com/vgl-hub/gfastats
GoaT CLI	0.2.5	https://github.com/genomehubs/goat-cli
Hifiasm	0.19.8-r603	https://github.com/chhylp123/hifiasm
HiGlass	44086069ee7d4d3f6f3f0012569789ec138f42b84aa44357826 c0b6753eb28de	https://github.com/higlass/higlass
MerquryFK	d00d98157618f4e8d1a9190026b19b471055b22e	https://github.com/thegenemyers/MERQURY.FK
Minimap2	2.24-r1122	https://github.com/lh3/minimap2
MitoHiFi	3	https://github.com/marcelauliano/MitoHiFi
MultiQC	1.14, 1.17, and 1.18	https://github.com/MultiQC/MultiQC
Nextflow	23.04.1	https://github.com/nextflow-io/nextflow
PretextView	0.2.5	https://github.com/sanger-tol/PretextView
purge_dups	1.2.5	https://github.com/dfguan/purge_dups
samtools	1.19.2	https://github.com/samtools/samtools
sanger-tol/ascc	-	https://github.com/sanger-tol/ascc
sanger-tol/ blobtoolkit	0.4.0	https://github.com/sanger-tol/blobtoolkit
Seqtk	1.3	https://github.com/lh3/seqtk
Singularity	3.9.0	https://github.com/sylabs/singularity
TreeVal	1.2.0	https://github.com/sanger-tol/treeval
YaHS	1.2a.2	https://github.com/c-zhou/yahs

### Wellcome Sanger Institute – Legal and Governance

The materials that have contributed to this genome note have been supplied by a Darwin Tree of Life Partner. The submission of materials by a Darwin Tree of Life Partner is subject to the **‘Darwin Tree of Life Project Sampling Code of Practice’**, which can be found in full on the Darwin Tree of Life website here. By agreeing with and signing up to the Sampling Code of Practice, the Darwin Tree of Life Partner agrees they will meet the legal and ethical requirements and standards set out within this document in respect of all samples acquired for, and supplied to, the Darwin Tree of Life Project.

Further, the Wellcome Sanger Institute employs a process whereby due diligence is carried out proportionate to the nature of the materials themselves, and the circumstances under which they have been/are to be collected and provided for use. The purpose of this is to address and mitigate any potential legal and/or ethical implications of receipt and use of the materials as part of the research project, and to ensure that in doing so we align with best practice wherever possible. The overarching areas of consideration are:

•   Ethical review of provenance and sourcing of the material

•   Legality of collection, transfer and use (national and international)

Each transfer of samples is further undertaken according to a Research Collaboration Agreement or Material Transfer Agreement entered into by the Darwin Tree of Life Partner, Genome Research Limited (operating as the Wellcome Sanger Institute), and in some circumstances other Darwin Tree of Life collaborators.

## Data Availability

European Nucleotide Archive: Omphiscola glabra (pond mud snail). Accession number PRJEB73399; https://identifiers.org/ena.embl/PRJEB73399. The genome sequence is released openly for reuse. The *Omphiscola glabra* genome sequencing initiative is part of the Darwin Tree of Life Project (PRJEB40665) and the Sanger Institute Tree of Life Programme (PRJEB43745). All raw sequence data and the assembly have been deposited in INSDC databases. Raw data and assembly accession identifiers are reported in Table 1 and Table 2.

## Author information

Members of the Wellcome Sanger Institute Tree of Life Management, Samples and Laboratory team are listed here: https://doi.org/10.5281/zenodo.12162482.

Members of Wellcome Sanger Institute Scientific Operations: Sequencing Operations are listed here: https://doi.org/10.5281/zenodo.12165051.

Members of the Wellcome Sanger Institute Tree of Life Core Informatics team are listed here: https://doi.org/10.5281/zenodo.12160324.

Members of the Tree of Life Core Informatics collective are listed here: https://doi.org/10.5281/zenodo.12205391.

Members of the Darwin Tree of Life Consortium are listed here: https://doi.org/10.5281/zenodo.4783558.
