# Supplementary material for: The genome sequence of the pond mud snail, Omphiscola glabra (O.F.Müller, 1774)
Source: Wellcome Open Res. 2025 May 20;10:261. [Version 1] doi: 10.12688/wellcomeopenres.24266.1 (PMC12553978; doi:10.12688/wellcomeopenres.24266.1)
Supplement: Peer review history

# Reviewer report: Reviewer response for version 1

**DOI:** 10.21956/wellcomeopenres.26763.r134470

**Published:** 2025-10-25

**Authors:** Alexander Fedosov

**Affiliations:** 1 Swedish Museum of Natural History, Stockholm, Stockholm, Sweden

I feel that this newly sequenced and assembled genome contributes to the growing body of genomic data on gastropods - a hyperdiverse, yet poorly studied from the genomic perspective lineage of invertebrates.

 Despite the clearly high conservation concern of the targeted species, I feel that there are other contexts that could be addressed in the introduction. Among them are the clearly too scarce available genomic resources for gastropoda altogether, whereas there are two high contiguity Lymnaeidae genomes published: *Biomphalaria glabrata*, and *Radix auricularia*. Availability of these resources could enable comparative genomic inferences in Hygrophyla, adding value to the present study. I have further more specific questions:

 The number of fragmented BUSCO genes wasn't reported.

 Which tissues were used for transcriptomics? If this was whole body, this should be stated clearly.

 It is clear from the introduction that the UK populations of the *Omphiscola glabra* have gone through periods of low population size - and so a bottleneck. Does the inferred heterozygosity value (0.45) compared to that in two related species with whole genome data available, reflect this bottleneck, or allows elaborating on how severe it was?

Are sufficient details of methods and materials provided to allow replication by others?

Yes

Is the rationale for creating the dataset(s) clearly described?

Yes

Are the datasets clearly presented in a useable and accessible format?

Yes

Are the protocols appropriate and is the work technically sound?

Yes

Reviewer Expertise:

Systematics and evolutionary biology of gastropods

I confirm that I have read this submission and believe that I have an appropriate level of expertise to confirm that it is of an acceptable scientific standard.

# Reviewer report: Reviewer response for version 1

**DOI:** 10.21956/wellcomeopenres.26763.r134479

**Published:** 2025-10-14

**Authors:** Grant Batzel

**Affiliations:** 1 University of California San Diego, San Diego, USA; 2 Center for Aquaculture Technologies, CA, USA

Genome assembly for a specimen of *Omphiscola glabra*, includes high coverage (57x) long-read PacBio Revio DNA sequencing, and HI-C sequencing to assemble into chromosome-level scaffolds (99.5% alignment to 18 chromosomes!). Further, RNAseq was conducted using Novaseq 6000 for annotation through EMBL pipeline.

 Overall, the stats on the assembly look really promising, and the sequencing data will be a great resource for other researchers doing population genetics studies on these animals, and others conducting comparative work. No major comments -- below are my minor suggestions.

 Sample acquisition and DNA barcoding:

 - Is there a cat# for the Ln2 shipper?

 - Any washing steps prior to flash freezing? 

 - Was the shell taken and preserved in collections as well?

 - Any idea on sex of the snails sampled?

 Nucleic acid extractions:

 - Cool that the MagAttract v2 protocol was so robust to snail mucus. Any special modifications for getting the protocol to work?

 - Is QC info (rin/din scores, qubit conc., nanodrop) of DNA/RNA material able to be posted as a table/supp. figure?

 Pacbio HIFI/RNA:

 - Noticed samples and libraries are plural. Preceding DNA/RNA extraction steps suggested the entire organism was sequenced as a whole. Were animals split into multiple libraries, and if so at what point in the workflow?

 - Were libraries sequenced on single flowcells for Pacbio and Illumina?

 - Amount of RNA used to make the library?

 - Is it possible to report any of the rna alignment stats or multiqc reports? A citation or url for the ensembl workflow could be helpful.

Are sufficient details of methods and materials provided to allow replication by others?

Yes

Is the rationale for creating the dataset(s) clearly described?

Yes

Are the datasets clearly presented in a useable and accessible format?

Yes

Are the protocols appropriate and is the work technically sound?

Yes

Reviewer Expertise:

evolutionary developmental biology

I confirm that I have read this submission and believe that I have an appropriate level of expertise to confirm that it is of an acceptable scientific standard.

# Reviewer report: Reviewer response for version 1

**DOI:** 10.21956/wellcomeopenres.26763.r124009

**Published:** 2025-06-20

**Authors:** Tauana Cunha

**Affiliations:** 1 Department of Biology, Loyola University Chicago (Ringgold ID: 2456), Chicago, Illinois, USA

Ruta & Taylor present a high-quality, chromosome-level genome for the threatened pond mud snail. The paper is a straightforward genome report. The genome is based on high coverage PacBio data with Hi-C scaffolding and a transcriptome for annotation. Below I explain my one major concern and other suggestions/questions to fill gaps and improve clarity of the paper.

 Main points:

 1. I have a concern about no voucher specimens being mentioned. Three individuals are listed, each with whole-body used to generate a different type of sequencing dataset. Was no other animal collected as a voucher reference for the data? It is highly desirable (and ideally required) to have a voucher in a permanent museum collection for future reference. There could be problems with specimen identification, species delimitation etc that could only be resolved in the future by knowing from which animal the molecular data was generated. In this particular case, the shells of each individual should have been deposited in a museum. Where are the shells/vouchers? Could you deposit them and share the voucher ID in the paper?

 2. Different individuals were used for the genomic data and for the Hi-C scaffolding data. How could that impact the assembly?

 3. Even if the genome was annotated externally, a summary of how that was done should be provided, so that readers can gauge the quality of annotation for themselves.

 4. When mentioning the EBP reference standard of 6.C.Q60, please provide some brief context to what that value and reference mean.

 5. Table 2 and text: it would be useful to also show BUSCO scores for the Metazoan dataset, since this is commonly used for many new genomes and would provide a better comparison across taxa. In the methods "three domain-level BUSCO lineages" are mentioned, but it is not clear which, and results for others are not shown.

 6. The first section header of the methods mentions DNA barcoding, which usually refers to sequencing COI, but nothing in that section relates to that. Perhaps change the header, or add the information about barcoding.

 7. The Library preparation section of the methods frequently refers to samples in the plural, but my understanding is that each library only had one source sample, so singular would be the correct here. In particular for the Hi-C paragraph, it is said that samples were amplified for 10-16 cycles, but this one sample was probably amplified with one specific number of cycles – the information should be specific for this sample/paper.

 Additional suggestions:

 8.Please add the reference for the data about the monitoring outcomes of reintroduced snails (second paragraph of background).

 9. On the section 'Genome sequence report, Sequencing data', the x for coverage is missing after 57.

 10. On the section 'Genome sequence report, Assembly statistics', please add a brief mention to how the genome was assembled, otherwise the reader has to refer back and forth to the methods while reading the results. Something like "The primary haplotype was assembled with Hifiasm and scaffolded with YaHS, and contigs…"

 11. Table 1: would be useful to have the coverage of each dataset in parentheses next to the Base count metrics.

 12. I assume the Agilent RNA 6000 Pico kit in the methods is from a Bioanalyzer?

 13. Figure 3 should have more clear axes labels, such as Coverage (PacBio? All sequencing data?) and GC Content.

 14. Adding the megabase length of chromosomes in the total assembly on the right axis of the Hi-C plot in Figure 5 would be a nice addition.

Are sufficient details of methods and materials provided to allow replication by others?

Partly

Is the rationale for creating the dataset(s) clearly described?

Yes

Are the datasets clearly presented in a useable and accessible format?

Yes

Are the protocols appropriate and is the work technically sound?

Yes

Reviewer Expertise:

Genomics, phylogenomics, gastropods, evolution

I confirm that I have read this submission and believe that I have an appropriate level of expertise to confirm that it is of an acceptable scientific standard, however I have significant reservations, as outlined above.
